# Alpha-mangostin inhibits intracellular fatty acid synthase and induces apoptosis in breast cancer cells

**DOI:** 10.1186/1476-4598-13-138

**Published:** 2014-06-03

**Authors:** Ping Li, Weixi Tian, Xiaofeng Ma

**Affiliations:** 1 College of Life Sciences, University of Chinese Academy of Sciences, No. 19A Yuquan Road, Beijing 100049, China

**Keywords:** Fatty acid synthase, Alpha-mangostin, Inhibitor, Breast cancer cells, Cell apoptosis

## Abstract

**Background:**

Fatty acid synthase (FAS) has been proven over-expressed in human breast cancer cells and consequently, has been recognized as a target for breast cancer treatment. Alpha-mangostin, a natural xanthone found in mangosteen pericarp, has a variety of biological activities, including anti-cancer effect. In our previous study, alpha-mangostin had been found both fast-binding and slow-binding inhibitions to FAS *in vitro*. This study was designed to investigate the activity of alpha-mangostin on intracellular FAS activity in FAS over-expressed human breast cancer cells, and to testify whether the anti-cancer activity of alpha-mangostin may be related to its inhibitory effect on FAS.

**Methods:**

We evaluated the cytotoxicity of alpha-mangostin in human breast cancer MCF-7 and MDA-MB-231 cells. Intracellular FAS activity was measured by a spectrophotometer at 340 nm of NADPH absorption. Cell Counting Kit assay was used to test the cell viability. Immunoblot analysis was performed to detect FAS expression level, intracellular fatty acid accumulation and cell signaling (FAK, ERK1/2 and AKT). Apoptotic effects were detected by flow cytometry and immunoblot analysis of PARP, Bax and Bcl-2. Small interfering RNA was used to down-regulate FAS expression and/or activity.

**Results:**

Alpha-mangostin could effectively suppress FAS expression and inhibit intracellular FAS activity, and result in decrease of intracellular fatty acid accumulation. It could also reduce cell viability, induce apoptosis in human breast cancer cells, increase in the levels of the PARP cleavage product, and attenuate the balance between anti-apoptotic and pro-apoptotic proteins of the Bcl-2 family. Moreover, alpha-mangostin inhibited the phosphorylation of FAK. However, the active forms of AKT, and ERK1/2 proteins were not involved in the changes of FAS expression induced by alpha-mangostin.

**Conclusions:**

Alpha-mangostin induced breast cancer cell apoptosis by inhibiting FAS, which provide a basis for the development of xanthone as an agent for breast cancer therapy.

## Introduction

Breast cancer is the most commonly diagnosed cancer and a leading cause of cancer death among women in both developed and developing nations. Furthermore, breast cancer has a low cure rate and easy recurrence, and always been treated with surgery. The most effective way is to search for drugs that could both induce apoptosis in cancer cell lines and delay tumor-growth.

Fatty acid synthase (FAS) is the key enzyme required for *de novo* synthesis of fatty acids [1]. It was discovered to play a role in breast cancer prognosis in 1994 when FAS was found as a molecular marker in breast cancer patients with a markedly worsened prognosis [2]. The high expression of FAS in human cancer and its association with poorer prognoses in breast [3], ovarian [4] and prostate carcinomas [5] suggest that high levels of FAS expression and activity provide an advantage for tumor growth and progression. This is different from the role of FAS-dependent fatty acid biosynthesis as an anabolic energy storage pathway in liver and adipose tissue. In fact, most human tissues express very low levels of FAS because endogenous fatty acid biosynthesis is down-regulated when a normal diet is consumed [6,7]. Interestingly, the differential expressions of FAS between cancer and normal tissues have led to the hypothesis that tumor-associated FAS could be exploited as a useful molecular target for the development of new therapeutic anti-metabolites [7,8]. Obstacle of FAS activity blocks tumor cell development, survival, aggressiveness and metastasis, and induces cell apoptosis in human cancer cells both *in vitro* and *in vivo*, however, has minimal effect on normal cells [8-11].

**Figure 1:**
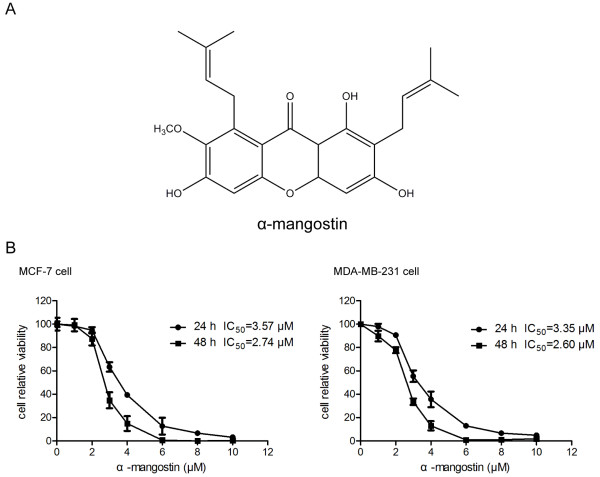
**Effect of α-mangostin on the viability of MCF-7 and MDA-MB-231 cells. A** Chemical structure of α-mangostin. **B** MCF-7 and MDA-MB-231 cells were treated with 0, 1, 2, 3, 4, 6, 8, and 10 μM α-mangostin for 24 h and 48 h. Cell viability was then determined by the CCK-8 assay as described in Materials and Methods. The percentage of cell viability was calculated as the ratio of α-mangostin treated cells to control cells. Data represented the mean ± SD of three independent experiments.

Mangosteen (*Garcinia mangostana* Linn) pericarp contains various phytochemicals, primarily xanthones, and has long been used for medicinal purposes in Southeast Asia [12]. Alpha-mangostin (α-mangostin, Figure 1A) is the most abundant xanthone existed in mangosteen pericarp. It has been confirmed to have anti-proliferative and apoptotic effects in various types of human cancer cells [12-16]. We previously reported that α-mangostin showed both fast-binding and slow-binding inhibitions to FAS *in vitro*. It inhibits FAS overall reaction with a half-inhibitory concentration (IC_50_) value of 5.54 μM noncompetitively with respect to NADPH, and partially competitively against both substrates acetyl-CoA and malonyl-CoA [17]. Its inhibitory activity is higher than classical FAS inhibitors such as C75, EGCG and curcumin.

Although it has become clearer that survival and proliferation of breast cancer cells are relay upon active FAS-catalyzed *de novo* fatty acid synthesis, the relationship between breast cancer-associated FAS hyperactivity and the efficacy of chemotherapy has not been studied. We hypothesized that the anti-cancer activity of α-mangostin related to its inhibitory effect on FAS, therefore we sought to determine whether α-mangostin exhibit anti-cancer activity through affecting intracellular fatty acid biosynthesis in breast cancer cells. We first examined how α-mangostin affects FAS expression level and activity in breast cancer cells, then the cytotoxicity of α-mangostin was investigated. We also investigated the possible pathways that involved in the modulation of FAS by α-mangostin, and found that α-mangostin could effectively suppress FAS expression and inhibit intracellular FAS activity, resulted in decrease of intracellular fatty acid accumulation. α-Mangostin could reduce cell viability and induce apoptosis in human breast cancer cells. Moreover, we found that α-mangostin would enhance its cytotoxicity on breast cancer cell after silence of FAS. These results, altogether, present the first evidence that α-mangostin induces cell apoptosis via suppressing FAS expression and inhibiting intracellular FAS activity.

## Materials and methods

### Materials

Acetyl-CoA, Malonyl-CoA, NADPH, DMSO, and α-mangostin were purchased from Sigma (St. Louis, MO, USA). Dulbecco’s modified Eagle’s medium (DMEM) and fetal bovine serum (FBS) were purchased from Life Technologies, Inc. (Gibco/BRL, Gaithersburg, MD). FAS antibody was obtained from BD Pharmingen (San Diego, CA, USA). FAK, phosphor-FAK^tyr397^, AKT, phospho-AKT^Ser473^, ERK1/2, phosphor-ERK1/2^Thr202/Tyr204^, Bax, Bcl-2, PARP and GAPDH were purchased from Cell Signaling Technology (Denvers, MA, USA).

### Cell lines and culture

The human breast epithelial cell lines MCF-7, estrogen receptor-positive cells derived from an in situ carcinoma, and MDA-MB-231, estrogen receptor-negative cells derived from a metastatic carcinoma, were used in the study. The cells were purchased from the American Type Culture Collection (ATCC; Rockville, MD, USA) and were grown in DMEM supplemented with 10% fetal bovine serum. Cells were maintained at 37°C in a humidified atmosphere of 95% air and 5% CO_2_.

### Cell viability assay

Cell viability was assessed by Cell Counting Kit (CCK-8; Dojindo Laboratories, Kumamoto, Japan) assay as previously described [18]. Briefly, cell were seeded at a concentration of 1 × 10^4^ cells/200 μl/well into 96-well plates, and allowed an overnight period for attachment. Medium was removed and fresh medium along with various concentrations of α-mangostin were added to cultures in parallel. Following treatment, drug-free medium (100 μl/well) and 10 μl CCK-8 solution were added and cells were incubated for 1 h at 37°C. The optical density (OD) value (absorbance) was measured at 450 nm by a microplate spectrophotometer (Multiskan, MK3). All experiments were performed in quadruple on three separate occasions.

### Analysis of apoptosis

Cell apoptosis detection was performed using an Annexin-V-FITC Apoptosis Detection Kit (BD company, US) according to the manufacturer’s protocol [18]. Briefly, cells were collected after 24 h treatment with α-mangostin. The cells were washed twice with cold PBS then resuspended in 1× binding buffer at a concentration of 1 × 10^6^ cells/ml. Then 500 μl cell suspension was incubated with 5 μl Annexin-V-FITC and 10 μl PI for 15 min in the dark and analyzed by a FACScalibur instrument (Becton Dickinson, San Jose, US) within 1 h. Apoptotic cells were those stained with Annexin V+/PI- (early apoptotic) plus Annexin V+/PI + (late apoptotic cell).

### FAS activity assay

After 24 h of exposure to α-mangostin, cells were harvested by treatment with trypsin-EDTA solution, pelleted by centrifugation, washed twice, and resuspended in cold phosphate buffered solution (PBS). Cells were sonicated at 4°C and centrifuge at 13,000 rpm for 30 min at 4°C to obtain particle-free supernatants. FAS activity was determined spectrophotometrically at 37°C by measuring the decrease of absorption at 340 nm due to oxidation of NADPH as previously described [19]. 50 μl Particle-free supernatant, 25 mM KH_2_PO_4_-K_2_HPO_4_ buffer, 0.25 mM EDTA, 0.25 mM dithiothreitol, 30 μM acetyl-CoA, 350 μM NADPH (pH 7.0) in a total volume of 500 μl were monitored at 340 nm for 100 s to measure background NADPH oxidation. After the addition of 100 μM of malonyl-CoA, the reaction was assayed for an additional 1 min to determine FAS–dependent oxidation of NADPH. FAS activity was expressed in nmoles NADPH oxidized min^-1^ mg protein^-1^.

### Immunoblot analysis

Following treatment of breast cells with α-mangostin at the corresponding concentration and for the indicated time, cells were harvested using trypsin-EDTA, washed twice with PBS, and stored at -80°C. Cells were lysed in lysis buffer (1 mM EDTA, 150 mM NaCl, 100 μg/ml phenylmethylsulfonyl fluoride 50 mM Tris–HCl, pH 7.5) for 30 min on ice and then a particle-free supernatant solution was obtained by centrifugation at 14,000 g for 15 min. All operations were at 0–4°C. A sample was taken for measurement of protein content by a bicinchoninic acid (BCA) assay (Pierce). Equal amounts of protein were heated in sodium dodecylsulphate (SDS) sample buffer (Laemmli) for 15 min at 95°C, separated on a 8% - 12% SDS-polyacrylamide gel and transferred onto polyvinylidene fluoride membranes. Membranes blocked with 5% nonfat milk powder (w/v) in TBST (10 mM Tris, 10 mM NaCl, 0.1% Tween 20) for 2–4 h at room temperature to prevent nonspecific antibody binding, and incubated with the corresponding primary antibody diluted in blocking buffer overnight at 4°C. After 3 × 10 min washes in TBS-T, blots were incubated for 1 h with corresponding peroxidase conjugated secondary antibody and developed employing a commercial kit (West Pico chemiluminescent substrate). Blots were reprobed with an antibody against GAPDH as control of protein loading and transfer.

### RNA interference

The sequence of the FAS-targeted siRNA (5′-TGGAGCGTATCTGTGAGAATT-3′) was reported previously [20]. As a nonspecific siRNA control, we used a scrambled-sequence siRNA duplex (5′ -UUCUCCGAACGUGUCACGUTT -3′). Both siRNAs were synthesized by Invitrogen. Breast cancer cells were transiently transfected with 20–40 nM siRNA-targeting FAS or scrambled siRNA in 60-mm dishes (5 × 10^5^ cells per dish) with the use of Lipofectamine 2000 reagent (Invitrogen) according to the manufacturer’s instructions. At the indicated time points after transfection, cells were used for analyses of FAS expression and activity.

### Quantification of fatty acid

After treatment with α-mangostin at the corresponding concentration for 24 h, cells were harvested using trypsin-EDTA, washed twice with PBS, and stored at -80°C. Intracellular fatty acid was determined with a Free Fatty Acid Quantification Kit (Bivision) according to the manufacturer’s instructions.

## Results

### The cytotoxicity of α-mangostin in breast cancer cells

To evaluate the cytotoxicity of α-mangostin, breast cancer cells were incubated with various concentrations of α-mangostin (0, 1, 2, 3, 4, 6, 8, 10 μM) for 24 and 48 h followed by a CCK-8 assay. As shown in Figure 1B, α-mangostin exhibited cytotoxic effects on MCF-7 and MDA-MB-231 cell growth in a dose- and time-dependent manner. The IC_50_ values of α-mangostin in MCF-7 cells were 3.57 μM in 24 h and 2.74 μM in 48 h. As for MDA-MB-231 cells, the IC_50_ values were 3.35 μM in 24 h and 2.26 μM in 48 h. No significant difference about cell viability was found between MCF-7 and MDA-MB-231 cells. These results suggested that α-mangostin could suppress growth and reduce the viability of breast cancer cells.

### α-Mangostin induced apoptosis in breast cancer cells

**Figure 2:**
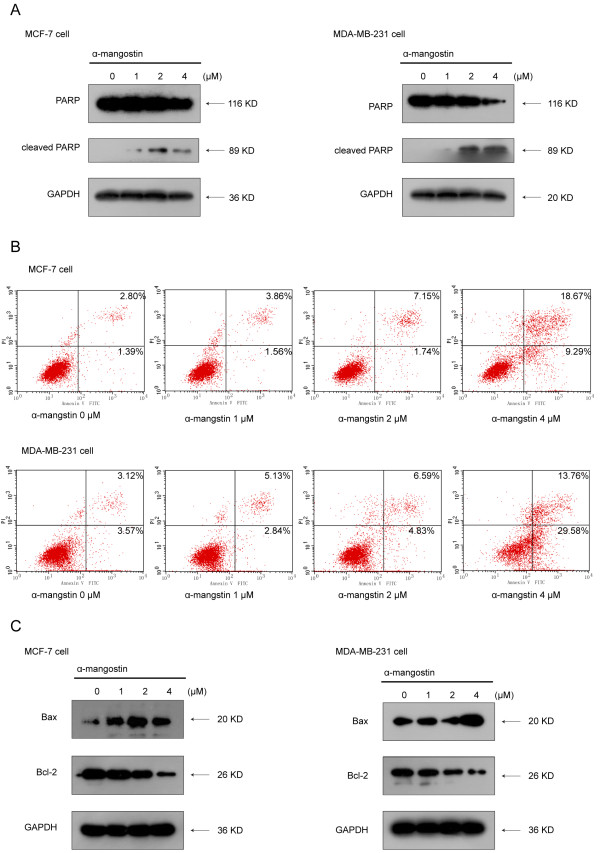
**Apoptotic effect of α-mangostin in MCF-7 and MDA-MB-231 cells. A** α-Mangostin induced apoptosis in MCF-7 and MDA-MB-231 cells as assessed by PARP cleavage (note intact PARP at 116 kDa and its cleavage product at 89 kDa) **B** MCF-7 and MDA-MB-231 cells were double-stained with annexin V and PI and analyzed by flow cytometry. The gate setting distinguished between living (bottom left), necrotic (top left), early apoptotic (bottom right), and late apoptotic (top right) cells. **C** The expression of Bax and Bcl-2 were determined by immunoblotting. MCF-7 and MDA-MB-231 cells were treated with α-mangostin at different concentrations for 24 h and equal amounts of lysates were immunoblotted with the corresponding antibody. Blots were reprobed for GAPDH as a loading control. In all cases, shown gels were representative of those obtained from at least two independent experiments.

To confirm whether the cell cytotoxicity induced by α-mangostin was related to apoptosis, we next measured PARP protein expression as markers of apoptosis. Treatment of MCF-7 and MDA-MB-231 cells for 24 h with α-mangostin induced a marked increase in the levels of the PARP cleavage product (89 kDa band) in a dose-dependent manner. To validate these results, the apoptotic effects of α-mangostin were also analyzed and quantified by flow cytometry using the Annexin V -FITC Apoptosis Detection Kit. As shown in Figure 2, α-mangostin induced breast cancer cells apoptosis in a dose-dependent manner, reaching very high levels at 4 μM concentration.

The Bcl-2 family of proteins regulates apoptosis and the gene products of Bcl-2 and Bax play important roles in apoptotic cell death. Therefore, we examined the effect of α-mangostin on Bcl-2 family proteins in MCF-7 and MDA-MB-231 cells. As shown by immunoblot analysis, breast cancer cells treated with α-mangostin result in decrease of anti-apoptotic Bcl-2 and a concomitant increase of pro-apoptotic Bax proteins, thereby caused a significant increase in the Bax/Bcl-2 ratio that favors apoptosis.

### α-Mangostin down-regulated expression and intracellular activity of FAS in breast cancer cells

**Figure 3:**
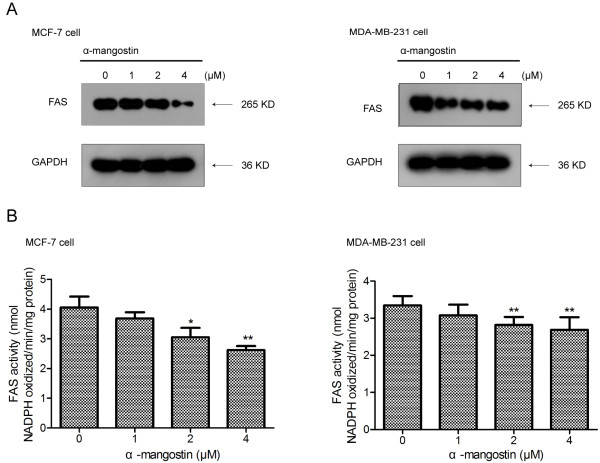
**α-Mangostin down-regulated FAS expression and inhibited intracellular FAS activity in breast cancer cells. A** Representative Western blots showing a dose-dependent inhibition of FAS expression levels in MCF-7 and MDA-MB-231 cells after treating with α-mangostin at 24 h. **B** Relative FAS activity was measured by spectrophotometrically monitoring oxidation of NADPH at 340 nm. Data were expressed as means ± SD (n = 3). *p < 0.05 compared to control (0 μM); **p < 0.01 compared to control (0 μM).

α-Mangostin preferentially suppressed growth and induced apoptosis of breast cancer cells. To clarify further mechanism, we proposed that there was a specific target for α-mangostin that may be activated in malignant cells. High level of FAS expression is considered one of the most common molecular changes in breast cancers. We therefore hypothesized that apoptosis of α-mangostin treated breast cancer cells was due to a decrease in FAS levels. Incubation of MCF-7 and MDA-MB-231 cells with the indicated concentrations of α-mangostin at 37°C for 24 h resulted in a dose-dependent inhibition of FAS expression (Figure 3A). We further checked the activity of FAS in cells treated with α-mangostin for 24 h as described in Materials and Methods, as a result, it reduced significantly in both MCF-7 and MDA-MB-231 cells (Figure 3B).

### α-Mangostin decreased intracellular fatty acid in breast cancer cells

As FAS plays a major role in the synthesis of phospholipids required for the newly synthesized cellular membrane in highly proliferating tumor cell, inhibition of FAS could reduce the amount of phospholipids and also decrease free fatty acid [21]. We employed free fatty acid quantification kit as described in Materials and Methods to measure intracellular fatty acid after treating with α-mangostin for 24 h and found that 4 μM α-mangostin evidently reduced intracellular fatty acid in both MCF-7 and MDA-MB-231 cells. This result further showed that α-mangostin suppressed both FAS expression and activity.

### α-Mangostin showed stronger cytotoxicity in breast cancer cells after silence of FAS

**Figure 4:**
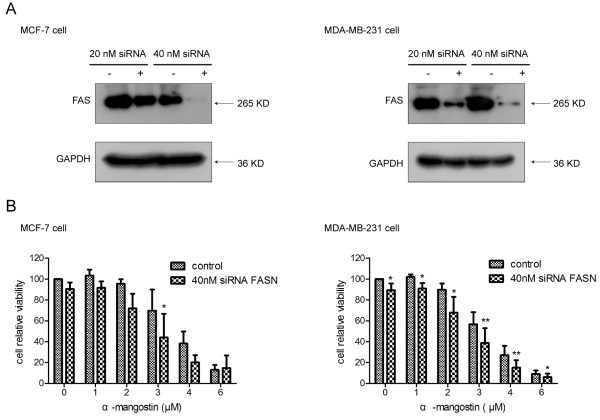
**Silence of FAS enhanced the cytotoxicity of α-mangostin in breast cancer cells. A** Breast cancer cells were transfected with siRNA-targeting FAS for 72 h. Equal amounts of total protein from MCF-7 and MDA-MB-231 cells transfected with siRNA-targeting FAS or with the control were subjected to immunoblotting analyses with antibodies against FAS or GAPDH. **B** Effect of siRNA-induced silencing of FAS expression on cytotoxicity induced by α-mangostin. Cytotoxicity of α-mangostin was enhanced by siRNA-induced reduction of FAS expression levels. Data represented the means ± SD of three independent experiments. *p < 0.05, **p < 0.01 compared with the same concentration of α-mangostin in control-transfected cells.

To specifically silence the expression of FAS, MCF-7 and MDA-MB-231 cells were separately transfected with siRNA-targeting FAS as described in Materials and Methods. As a control for specificity of RNAi, cells were transfected with a non-specific control pool of RNAi. As shown Figure 4A, FAS RNAi at 40 nM severely suppressed constitutive FAS over-expression in MCF-7 and MDA-MB-231 when compared to control cells transfection with non-specific RNAi and FAS RNAi at 20 nM.To assess potential effects of α-mangostin on FAS knockdown breast cancer cells, FAS RNAi-transfected and negative control breast cancer cells were harvested, re-cultured in 96-well plates and cell viability was judged using a CCK-8 assay after exposure to various concentrations of α-mangostin. We found that α-mangostin showed stronger toxicity on FAS RNAi-transfected cells compared with nonspecific control pool of RNAi (Figure 4B), which was different from C75, a classical FAS inhibitor, which had been reported that could rescue cytotoxicity after knockdown of FAS.

### α-Mangostin inhibited PI3K/AKT signaling pathway but activated MAPK/ERK1/2 signaling pathway

**Figure 5:**
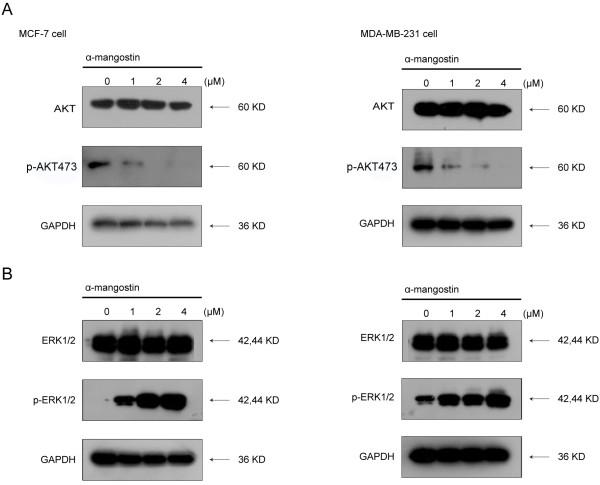
**Effects of α-mangostin on PI3K/AKT and MAPK/ERK1/2 signaling pathways in breast cancer cells. A** α-Mangostin induced FAS blockade led to down-regulate p-AKT in both MCF-7 and MDA-MB-231 cells. **B** α-Mangostin induced ERK1/2 activation in both MCF-7 and MDA-MB-231 cells. Cells were treated with α-mangostin at different concentrations for 24 h and equal amounts of lysates were immunoblotted with the corresponding antibody. Blots were reprobed for GAPDH as a loading control. In all cases, shown gels were representative of those obtained from at least two independent experiments.

Complicated signaling pathways are involved in the regulation of FAS expression and the two well-studied major pathways are the mitogen-activated protein (MAPK)/ERK cascades and PI3K/AKT pathways in breast cancer cells [22]. MAPK/ERK1/2 and PI3K/AKT were thought to play important roles in breast cancer cell, which promote cellular proliferation, cellular survival, and anti-apoptotic responses. To assess whether the activities of ERK1/2 and AKT were affected by α-mangostin, we explored the phosphorylation of ERK1/2 and AKT in MCF-7 and MDA-MB-231 cells after treatment with α-mangostin (0–4 μM) for 24 h. Immunoblot analysis with anti-phospho-specific antibody was then performed. The results showed that phosphorylated forms of AKT (p-AKT) were noticeably decreased when treated with α-mangostin in MCF-7 and MDA-MB-231 cells (Figure 5A). Interestingly, increased levels of active ERK1/2 were detected in MCF-7 and MDA-MB-231 cells exposed to increasing concentrations of α-mangostin (Figure 5B). Moreover, the total levels of the corresponding proteins (ERK1/2, and AKT) were not altered in both of the two breast cancer cells. Our results demonstrate that PI3K/AKT signaling pathway may relate to the down-regulation of FAS expression by α-mangostin.

### α-Mangostin down-regulated phosphorylation of FAK in breast cancer cells

**Figure 6:**
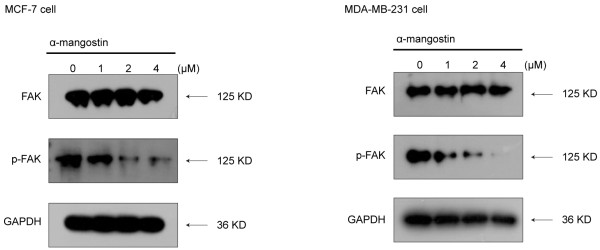
**α-Mangostin down-regulated phosphorylation of FAK in breast cancer cells.** Tyr^397^ phosphorylation of FAK in both MCF-7 and MDA-MB-231 cells were decreased after treated with α-mangostin at different concentrations for 24 h. Equal amounts of lysates were immunoblotted with the corresponding antibody. Blots were reprobed for GAPDH as a loading control. In all cases, shown gels were representative of those obtained from at least two independent experiments.

It was reported that proteins differentially abundant across human breast cancer cell line models were associated with FAK expression levels and especially in MCF-7 and MDA-MB-231 cells FAK was over-expressed. Tyr^397^ phosphorylation of FAK was a critical step for obtaining FAK activity. As shown in Figure 6, MCF-7 and MDA-MB-231 cells treated with α-mangostin for 24 h resulted in rapid decreases in Tyr^397^ phosphorylation of FAK. Furthermore, we found that Tyr^397^ phosphorylation of FAK was down-regulated after silence of FAS.

## Discussion

Breast cancer is a deadly disease that continues to disrupt the lives of millions of women and their families worldwide, and it is the second leading cause of cancer induced deaths for women in the United States, where one in eight women are affected by breast cancer. These statistics are frightening despite decades of innovative research that led to the development of newer targeted therapies.

Owing to the differential expression levels between cancer and normal cells, FAS has been suggested as a potential molecular target for anticancer drug development [9,23-25]. Given the widespread high expression of FAS in many types of human cancer [9], combinations of novel drugs directed against FAS-dependent endogenous fatty acid biosynthesis may provide increased efficacy for treating common human cancer [26]. α-Mangostin is the most abundant and one of the most studied xanthones due to its potential therapeutic properties. It has long been suggested that α-mangostin possess anti-cancer properties in prostate [27], breast [28], lung [29], and colorectal [30] cancers, through initiation of apoptosis through the regulation of cell death pathways, suppression of cancer cell proliferation and metastasis via inhibition of anti-apoptotic molecules and cell cycle arrest [31]. In our previous study, we have demonstrated that α-mangostin was a FAS inhibitor with stronger inhibitory than classical FAS inhibitors. Our present study provides the first evidence that the anti-cancer effect of α-mangostin is, at least partly, related to its inhibitory activity on FAS.

Considering that α-mangostin could induce apoptosis in breast cancer cells and we have demonstrated that α-mangostin inhibit FAS *in vitro*, we proposed, and subsequently set out to prove that α-mangostin induced cancer cells apoptosis by inhibiting FAS.

Our data on the apoptotic effects of α-mangostin in breast cancer cells support the well-investigated properties of α-mangostin as a potent anti-cancer candidate. We found that α-mangostin could induce apoptosis by increasing the level of the PARP cleavage product, up-regulating the level of anti-apoptotic protein Bcl-2 and down-regulating pro-apoptotic protein Bax. The balance between anti-apoptotic and pro-apoptotic proteins of the Bcl-2 family has been shown to play a critical role in maintaining cell survival.

**Figure 7:**
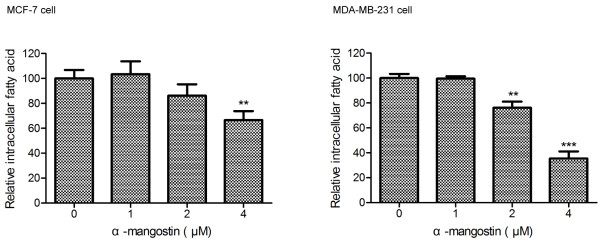
**Inhibitory effect of α-mangostin on the amount of intracellular fatty acid.** Intracellular free fatty acid was detected by Free Fatty Acid Quantification Kit. Data were expressed as means ± SD (n = 3). The experiments were repeated twice. **p < 0.01 significantly different from control (0 μM); ***p < 0.001 significantly different from control (0 μM).

Up to now, no previous studies have evaluated the influence of α-mangostin on intracellular FAS expression and activity or further effect on free fatty acids in breast cancer cells. FAS is a metabolic enzyme involved in the synthesis of long-chain saturated fatty acids that are essential for membrane establishment in proliferating cells. In many types of cancer cells, including breast cancer cells, over-expression of FAS robustly induces *de novo* lipogenesis. The generated lipids are integrated into membrane lipid rafts and modulate membrane receptor tyrosine kinases (such as the EGFR family) which, in turn, results in the initiation of oncogenic signaling pathways involved in cell survival, proliferation, migration and invasion [32,33]. Up-regulation of FAS activity in cancer cells is significant for the synthesis of phospholipids, which are necessary for the *de novo* establishment of cellular membrane in highly proliferating tumor cells [34]. Thus, once FAS activity was inhibited, the phospholipids synthesis was blocked, cancer cell proliferation decreased and ultimate apoptosis. Furthermore, the inhibition of FAS could also induce changes in the synthesis of membrane phospholipids and subsequently assembling of lipid rafts, as well impair the proper localization of EGFR to the cell membrane in breast cancer cells [35].Considering the main function of FAS is catalyzing the biosynthesis of long chain fatty acid, we determined the intracellular free fatty acid by Free Fatty Acid Quantification Kit. As Figure 7 shown, α-mangostin could reduce the amount of fatty acid dose-dependently in MCF-7 and MDA-MB-231 cells, which indicated that α-mangostin did inhibit FAS function.

It is apparent that FAS occupies an important niche in tumor cells. In this study, we found that α-mangostin, similar to C75, could decrease the amount of intracellular free fatty acid. Here our study confirmed that α-mangostin induced cytotoxicity to reduce FAS expression and inhibit intracellular FAS activity in both MCF-7 and MDA-MB-231 cells.

Prior studies have reported that the sensitivity of breast cancer cell to cytotoxicity induced by cerulenin or C75 was closely correlated with constitutive FAS expression [19,36]. However, no corresponding data of α-mangostin have been reported. The observation that siRNA-mediated FAS silencing enhanced the cytotoxicity of α-mangostin in breast cancer cells was unexpected. This result was in contrast with that of C75, a synthetic FAS inhibitor, which could restore cytotoxicity after knock down FAS gene [19]. The result may be explained by the reduction of free fatty acid in α-mangostin treated cells, which indicated that when both FAS expression and activity were inhibited, the cell viability was dramatically decreased. This explains that FAS play an important role in maintaining cell viability and growth.

PI3K/AKT and MAPK/ERK1/2 signaling pathways have been implicated in the regulation of FAS expression in breast cancer cells [22] and other cells [37-39]. Down-regulate p-AKT or ERK1/2 could attenuate FAS expression. So we determined the effect of α-mangostin on these two pathways. We found that α-mangostin could down-regulate p-AKT in a dose-dependent manner in both MDA-MB-231 and MCF-7 cells. As for ERK1/2, α-mangostin could up-regulate the active p-ERK1/2 levels in both MDA-MB-231 and MCF-7 cells. These results demonstrated that the reduction of FAS expression induced by α-mangostin was via PI3K/AKT signaling pathway.

Although the rate of mortality from breast cancer has decreased in developed countries, the incidence of breast cancer has actually risen. It is estimated that more than 90% cancer-related deaths are due, directly or indirectly, to cancer metastasis. The process of metastasis and invasion of tumor cells are associated with FAK [40,41], which functions as a major mediator that translate extracellular, integrins, growth factor-mediated signaling events to the intracellular environment, typically via PI3K/AKT and MAPK-ERK1/2 signaling cascades [42-46]. FAK activation is mediated by autophosphorylation at tyrosine-397. There are modest increases of FAK in MCF-7 and MDA-MB-231 cells relative to normal cells.

The anti-metastatic effect of α-mangostin was found in many cancer cells [28,29]. And researchers have found that inhibition of FAS could attenuate FAK [47]. In the present work, we found that α-mangostin could down-regulate Tyr^397^ phosphorylation of FAK in both two breast cancer cells. So we could draw a conclusion that the anti-metastatic effect of α-mangostin may be related with its inhibitory effect on FAS. Considering FAK is necessary for tumor invasion and metastasis. Further investigation with related study designs will be necessary to confirm and further examine the associations observed in our study.

Until fairly recently, breast cancer was treated as a single deadly disease for which the most extreme treatments were justified [48]. The search for novel drugs is still a priority goal for breast cancer therapy, due to the rapid development of resistance to chemotherapeutic drugs. In addition, the high toxicity usually associated with some cancer chemotherapy drugs and their undesirable side-effects increase the demand for novel anti-cancer drugs active against breast cancers, with fewer side-effects and/or with greater therapeutic efficiency. As a natural compound, α-mangostin is a prenylated xanthone isolated from the fruit hull of *Garcinia mangostana* L., which is a tropical fruit native to Southeast Asia that is often referred to as “the queen of fruits”. Products containing mangosteen fruits represented the sixth highest selling single-herb dietary supplement in US, in 2008 with sales exceeding $ 200 million [49]. It was reported that consumption of a semi-purified diet with 845 mg/kg α-mangostin showed no adverse effect in mice [50]. This suggests that α-mangostin is a promising agent for the chemoprevention of breast cancers, because it is a dietary component, relatively non-toxic, inexpensive, and consumption of α-mangostin can be easily adopted into the lifestyles of most women. Although the human bioavailability of α-mangostin is not clear, studies have revealed the bioavailability and metabolism of α-mangostin in mice and rats [51,52]. These studies could provide some useful information to the bioavailability of α-mangostin in human body.

It is worth mentioning that as a FAS inhibitor, α-mangostin was proven to reduce the viability of FAS over-expressed adipocytes. However, the cytotoxicity of α-mangostin on adipocytes was much less than that of breast cancer cells. So if we choose appropriate concentrations, α-mangostin could treat breast cancer targeting FAS without affecting adipocytes.

In summary, our current study demonstrated that α-mangostin, a potent FAS inhibitor, down-regulated FAS expression, inhibited intracellular FAS activity, decreased the amount of intracellular fatty acid and thus induced apoptosis in human breast cancer cells. Based on our finding, we suggest that the anti-cancer activity of α-mangostin might be through inhibiting fatty acid biosynthesis in cancer cells. Therefore, we conclude that its inhibitory effect on FAS has a therapeutic potential, giving a novel means of controlling breast cancer. This compound could be further explored for understanding the action mechanisms and *in vivo* model to justify if effective for prevention and treatment of breast cancer.

## Abbreviations

CCK-8: Cell counting kit; DMEM: Dulbecco’s modified Eagle’s medium; FAS: Fatty acid synthase; FBS: Fetal bovine serum; IC_50_: The half inhibition concentration; p-AKT: Phosphorylated-AKT.

## Competing interests

All authors declare they have no competing interests.

## Authors’ contributions

XM, as the principal investigator, was responsible for the concept and design of the study. XM, PL conducted the research and wrote the manuscript. WT provided expertise in enzymology analysis. PL did the whole experiments of the study. All authors participated in the preparation of, and have approved the final version of the manuscript.
